# Progressing from Simple to Multidimensional Models Towards a Biopsychosocial Framework of Addiction

**DOI:** 10.1007/s40429-026-00752-0

**Published:** 2026-06-11

**Authors:** Paul S. Regier

**Affiliations:** Department of Psychiatry, University of Pennsylvania, Philadelphia, PA 19104 USA

**Keywords:** Dopamine, Inhibition, Allostasis, Incentive Salience, Biopsychosocial, Psychedelics

## Abstract

**Purpose of Review:**

Neuroscientific models of addiction have evolved over the past four decades from single-circuit dopamine frameworks to dual-process, tripartite, and multidimensional accounts. Despite these advances, existing models incompletely account for the lived experience of addiction and for developmental, social, and mental-health factors that contribute to heterogeneity in risk and outcome. This review synthesizes this progression and evaluates the need for more integrative frameworks.

**Recent Findings:**

Recent work highlights addiction-related alterations in large-scale brain networks and demonstrates that psychosocial and contextual factors play a more central mechanistic role than previously acknowledged. Emerging interventions, including psychedelic-assisted therapies, further underscore the relevance of multilevel change across neurobiological, cognitive, and affective domains.

**Summary:**

Framing addiction as a multilevel, interactive process within a biopsychosocial framework provides a unifying perspective that accommodates heterogeneity in pathways and outcomes. Integrative models may better inform mechanistic research and guide the development of more precise, clinically meaningful interventions.

## Introduction

The problem of drug addiction is a significant one, causing societal, economic, familial, and personal harms [1, 2]. In the last ten years, use of substances in the United States has become more hazardous, with drug supplies often contaminated with high-potency opioids [3] and animal tranquilizers [4, 5]. Overdose rates prior to the introduction of fentanyl were steadily rising, slowly; however, after fentanyl was introduced, overdose rates spiked sharply [6, 7], from the thousands to hundreds of thousands over the course of a decade.

There are only approved pharmacological interventions for some substance use disorders (SUDs), including agonist and partial agonist treatments for opioid and nicotine-use disorder as well as opioid antagonist treatment for alcohol- and opioid-use disorder. These type of treatments for addiction not only reduce drug use and improve treatment adherence [8], they also help to prevent fatal overdose and reduce risk of all-cause mortality broadly [9, 10]. Other SUDs like stimulant and cannabis-use disorder remain without any pharmacological treatments.

Treating the biological basis of a disorder, which is primarily neurological in substance use disorders, bolsters opportunities for change, allowing for new associations to be made. Addiction is not only a neurobiological disorder but also a process of learning and unlearning associations between subjective emotional experiences and environmental cues that drive behavior [11]. Individuals with addiction may exhibit behaviors that can seem counter-intuitive to the observer and counter-productive to the user, but research suggests drug-use behavior can be thought of as an inflexible decision-making process [12, 13], with actions that are rational within an behavioral economic framework [14, 15], and with structural and functional changes in the brain that underlie decisions to use drugs [16]. Research suggests many possible causes of severity, and pathways to recovery, with lived experience containing within-person, internally-valid reasons for drug-use behaviors [17–20].

Decades of research using animal models and circuit-based approaches have identified underlying neural correlates of addiction [2]; however, these discoveries have struggled to create novel, effective interventions [21, 22]. One of the issues has been a hyperfocus on the dopamine system and its maladaptation as the primary mechanism driving addictive behavior [23]. Even if this circuit were the sole driver of addiction, historical attempts to modify it have been largely unsuccessful, in part because this system is vital for many natural functions [24, 25]. Of course, dopamine plays a key role in the development and maintenance of addiction; however, there’s a lack of integrative models defining the problem of human addiction from a biopsychosocial perspective, accounting for developmental, social, psychological, neurocognitive, and biological factors.

## Psychosocial Factors

On average, there are noted differences in environmental factors between those with addiction and the general population [26], with the former having grown up with more childhood maltreatment [27–29], lower socioeconomic status [30], and more dysfunctional social and family structures [31]. One of the early studies to recognize the role of environment in increasing risk for problematic drug use was the Adverse Childhood Experiences (ACE) Study [27]. Since then, prior adversity has been increasingly recognized as an over-represented factor in drug addiction [28, 29], suggesting a role of trauma affecting development in a way that increases addiction risk [32], or perhaps a role in substance use “managing” symptoms related to trauma and mental health [33]. Factors like trauma and SES often occur during childhood, but many of these factors carry over into adulthood. Current living situations are often worse for those with addiction, with higher rates of homelessness and unstable housing, lower employment and income, and higher exposure to violence and victimization [34–36].

Mental health disorders, including depression, anxiety, and PTSD are other critical factors that co-occur with SUDs [37–40]. Epidemiologic evidence supports elevated risk for SUD onset and progression among those with prior mental disorders [41]; however, patterns from large national surveys suggest nearly half of those with SUD exhibit some form of mental illness, but only one-third of those with mental illness exhibit SUD [42]. Mental health and drug addiction are difficult to disentangle, as symptoms of these disorders often overlap with the those of drug addiction, complicating attribution of impairment to one disorder or another. Treatment initiation and adherence are concerns in both SUD and mental illness, with rates ranging from 25% of those with SUD and 60% with depression seeking treatment, and approximately only one-half remaining in treatment after six months [42–46].

Substance use behaviors and psychosocial factors converge in their impact on neurocognitive processes that support valuation, learning, and regulatory control, shaping decision-making. Overlapping patterns have been observed, with noted differences in executive function, memory, and social cognition [12, 47–49]. Underlying these neurocognitive and decision-making challenges are distinct patterns of neural changes. Trauma affects stress systems, interacting with dopamine system changes during development, increasing risk of addiction [32]. Mood disorders have been associated with large-scale brain network changes, such as an overactive default-mode network (DMN) [50], and severe symptoms can result in drug misuse to cope with excessive worry and rumination. Escalated substance use, resulting in increased tolerance and subsequent withdrawal, often exacerbate mental health symptoms and underlying neural processes [51]. Depending on the primary source of dysfunction (e.g., addiction, mental health), these cognitive and underlying neural patterns may differentially impact outcomes and recovery trajectories [52].

### The Dopamine Model

In the mid to late 20th century, prominent models emphasized dopamine’s hedonic properties and dopamine depletion [53, 54]. Subsequent research identified this circuit as the mesolimbic pathway, with dopaminergic neurons projecting from ventral tegmental area to ventral striatum, including the nucleus accumbens [55]. The nucleus accumbens is critical for reinforcement-related learning and the motivational impact of salient cues [56], and dopaminergic signaling supports adaptive approach behavior in natural reward contexts [57, 58].

The incentive salience model represents one of the frameworks that clarified dopaminergic mechanisms underlying the development and maintenance of addiction. Berridge and Robinson [59] found that motivational drive for reward (‘wanting’) could be dissociated from hedonic experience (‘liking’) [60], with separable underlying mechanisms: dopamine for wanting and the endogenous opioid system for liking [61]. Parallel work further clarified dopamine’s role in reward learning through reward prediction error signaling, whereby dopamine responses initially track unexpected reward and subsequently shift to cues that predict reward availability [62, 63]. Together these findings provide a mechanism by which repeated drug exposure can result in preferential incentive salience for drug cues, biasing behavior towards drug use over natural rewards.

These seminal studies and theories provided a fundamental cornerstone of drug addiction and at the same time led to increased focus on dopamine as both primary mechanism and therapeutic target [23, 64]. This is a problem that is not unique to addiction, as it and other bench to bedside research has not produced validated interventions as expected [65]. Although animal models of addiction, such as self-administration and conditioned place preference, identified numerous pharmacological agents capable of reducing drug use and relapse in animals, these compounds showed limited translational validity for complex human addiction phenotypes [22, 23, 64]. Limitations arose in part from the models themselves, as single-choice paradigms proved less predictive than those incorporating alternative reinforcers, and from the difficulty of intervening at subcortical circuits essential for motivation and survival [66, 67]. Though preclinical models with dopamine agonists and antagonists showed great promise, trials in humans were largely unsuccessful and sometimes led to worse conditions [68–70]. Beyond the addiction problem, the challenge of using dopamine agents is also illustrated clinically by treatment of schizophrenia with dopamine antagonists, which is associated with diminished motivation and self-care [24, 71].

Moreover, much of the foundational dopamine literature was derived from stimulant research, in which cocaine and amphetamines directly elevate synaptic dopamine. Other substances primarily act on other neurotransmitter systems, such as opioids acting on the µ-opioid systems, which in turn interact with the dopamine system [72], implicating differential reward circuit adaptation [64]. In parallel, accumulating evidence indicates that drug addiction behaviors reflect broader dysfunction across corticostriatal and limbic circuitry, as well as large-scale networks involved in salience attribution, craving, and self-referential processing [16, 73].

Thus, the prominence of dopamine-centered explanations reflects both their empirical success in controlled settings and their limitations when applied to the heterogeneity and complexity of human addiction. While they remained a critical component of addictive behavior, explanatory frameworks needed to extend beyond dopaminergic circuits to incorporate regulatory control, affective state, and contextual influences.

## The Imbalance of Bottom-Up and Top-Down

Evolutions of the dopamine model included additional processes that either modulated or disinhibited a reward system affected by chronic drug use. Two notable examples are dual-process models that were introduced in the last two decades. Goldstein and Volkow’s iRISA model emphasizes impaired response inhibition alongside aberrant salience attribution to drug cues [74, 75]. Koob’s allostasis model, adapted from the opponent-process theory, emphasizes negative reinforcement and stress-system recruitment over repeated drug exposure [76, 77].

The dopaminergic system interacts with the stress system, and the allostasis model suggests a type of negative feedback loop formed between parts of the ventral striatum with the extended amygdala and locus coeruleus [78]. Over time, drug use results in a dampened positive response, offset by a heightened negative counter-process regulated by homeostatic mechanisms. This negative process comes on more slowly and results in negative after effects like withdrawal, which facilitates negative reinforcement, or the removal of the negative effect with more drug use [76, 77, 79].

The dopamine system also sends and receives reciprocal projections from circuits within the prefrontal cortex, involved in top-down regulation of subcortical processes governing emotional and reward signals [80]. The iRISA model suggests compulsive use is driven by dysregulated prefrontal and other regulatory regions (e.g., orbitofrontal and anterior cingulate cortices) rewired in a way that prioritizes drug rewards with decreased cognitive processes dedicated to natural rewards. The model suggests that prefrontal dysregulation explains other neurocognitive impairment like learning and memory issues, attentional deficits, valuation, and self-awareness [48].

These two models clearly expanded the understanding of addiction as more than a disrupted dopaminergic process; however, each accounted for distinct and partially dissociable components of compulsive drug use. Their integration into a unified model, described next, represented an important conceptual advance; a single model that explains the cycle of maladaptive drug seeking behavior.

## The Addiction Cycle

Building on the complementary insights offered by iRISA and allostatic models, Goldstein, Koob, and Volkow proposed a tripartite framework commonly referred to as the *addiction cycle* [81]. This model integrates three interacting phases: (1) binge/intoxication, (2) withdrawal/negative affect, and (3) preoccupation/anticipation, each emphasizing partially distinct but interacting neural systems. Importantly, the cycle provided a unifying account of how reward-driven, stress-driven, and regulatory failures occur at different phases of the addiction cycle, facilitating sustained drug use.

The binge/intoxication phase is initiated and maintained by heightened incentive salience [25], whereby drug-related cues acquire disproportionate motivational value relative to natural rewards. This process is supported by mesolimbic and corticostriatal circuitry that biases learning and valuation toward substance-related stimuli, promoting repeated use even with a diminishment of positive results. As incentive salience increases, behavior becomes progressively cue-driven and less sensitive to long-term consequences.

With repeated use, the model transitions to the withdrawal/negative affect phase, in which stress and aversive systems are recruited as a consequence of neuroadaptations associated with chronic exposure. Circuitry involving the extended amygdala and related stress-responsive regions becomes increasingly engaged, producing dysphoria, irritability, and heightened negative affect. Drug use during this stage is increasingly motivated by negative reinforcement, the alleviation of aversive internal states, rather than the pursuit of positive reward.

The final phase, preoccupation/anticipation, reflects failures of regulatory control that undermine attempts to abstain. Dysfunction within prefrontal and cingulate control networks compromises inhibitory control, self-monitoring, and flexible decision-making, allowing cue and stress-induced craving to precipitate relapse. In this stage, top-down regulation is insufficient to counteract the combined motivational pull of incentive salience and negative affect, effectively restarting the cycle. Repeated cycles of intoxication and withdrawal are accompanied by experience-dependent neuroplastic changes within reward, stress, and regulatory circuitry [81, 82]. Driven by glutamatergic mechanisms, these adaptations consolidate cue-driven learning and modulate affective systems, contributing to the persistence of drug-seeking behavior and reduced behavioral flexibility over time [83].

The addiction cycle represents a significant conceptual advance by integrating incentive-driven reward processes, stress-related negative reinforcement, and regulatory failure within a single temporal framework. However, while the model captures core neural dynamics sustaining addiction, it remains primarily circuit-focused and does not fully account for developmental history, psychiatric comorbidity, or social context. These limitations motivate further expansion toward models capable of accommodating individual differences in pathway dominance and broader biopsychosocial influences.

## The Biopsychosocial Framework

The biopsychosocial framework provides a structure for understanding addiction as an emergent condition arising from interacting biological, psychological, and social processes [84, 85]. Instead of a single mechanistic pathway, the framework organizes how multiple processes unfold and interact over time. Within this perspective, addiction is characterized as a chronic, relapsing disorder of the brain while acknowledging that addictive behavior emerges from an integration of neural, psychological, and contextual influences rather than from neural processes alone. This framework also accommodates multi-determinant pathways [86], such that different individuals may develop and maintain drug addiction through distinct configurations of interacting factors and vulnerabilities.

**Fig. 1:**
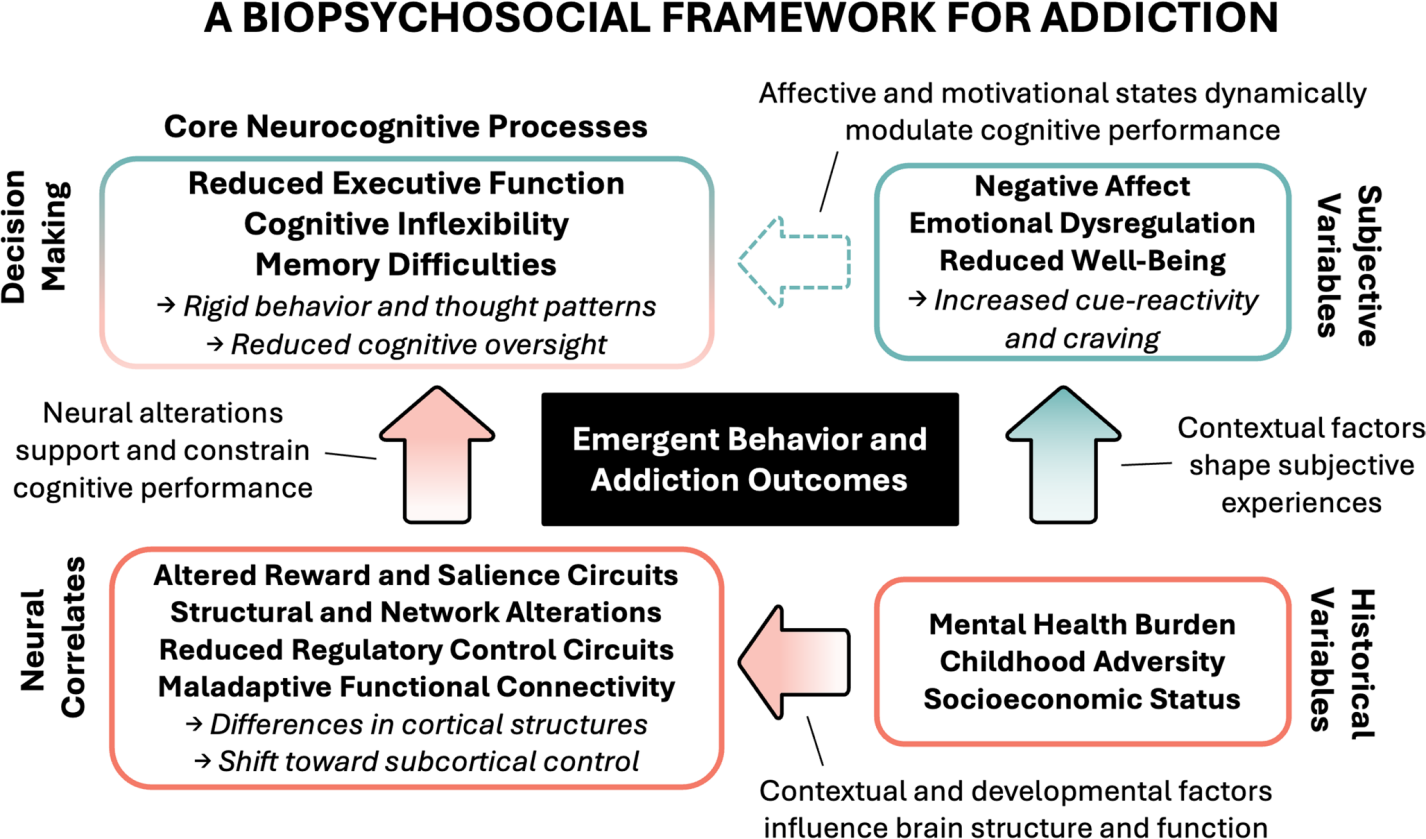
Addiction is conceptualized as an emergent condition arising from interactions among contextual factors, neural systems, core neurocognitive processes, and subjective states. Contextual variables (e.g., childhood adversity, socioeconomic status, and mental health burden) influence brain structure and function, contributing to alterations in reward and salience circuits, regulatory control systems, and large-scale functional connectivity. These neural alterations support and constrain core neurocognitive processes, including executive function, cognitive flexibility, and memory, which serve as a proximal interface for decision-making and behavior. Affective and motivational states (e.g., negative affect and emotional dysregulation) dynamically modulate cognitive performance, influencing behavior in real time. Illustrative pathways are depicted: one pathway (red/orange) reflects longer-term contextual and developmental influences on brain structure and regulatory balance, contributing to shifts toward subcortical influence and persistent changes in decision-making; a second pathway (blue/green) reflects the influence of affective and regulatory states on moment-to-moment cognitive performance. These pathways are not exhaustive but represent examples of how interacting processes across levels may give rise to heterogeneous addiction trajectories

The interaction of environmental factors and mental health burden provides an example of how pathways may unfold within this framework. Early-life adversity, including trauma and socioeconomic disadvantage, can influence the development of neural systems involved in stress regulation, reward processing, and cognitive control. These changes are associated with alterations in cortical and hippocampal structure, as well as shifts toward greater input from subcortical stress-related circuitry relative to cortical regulatory systems [87]. Such neurobiological adaptations can impair cognitive processes including flexibility, inhibition, and memory, while co-occurring subjective emotional states further disrupt cognitive control. Together, these interacting processes can contribute to maladaptive decision-making patterns, promoting substance use as a means of regulating affect and reinforcing continued use over time. Figure 1 illustrates one such pathway, though multiple configurations are possible. Table 1 defines the constructs that comprise the example pathway. 

**Table 1:** Examples of factors and components that interact with neural correlates to influence cognition and behavior

Factor	Construct	Definition	Core Components	Relevance to Addiction
Neurocognition *(Output)*	**Executive Function**	Top-down cognitive control processes that regulate goal-directed behavior	Inhibition, attention, working memory	Deficits contribute to impulsivity and reduced behavioral control
	**Cognitive Flexibility**	Ability to adapt thoughts and behavior based on changing contingencies	Set-shifting, updating, reversal learning	Impairments contribute to rigid behavior and perseveration
	**Episodic Memory**	Memory for personal experiences and contextual information guiding future decisions	Encoding, consolidation, retrieval	Deficits contribute to myopic planning and decision-making
Environmental/Developmental *(Historical)*	**Childhood Adversity**	Early-life exposure to stress or trauma	Abuse, neglect, parental challenges	Shapes neural development and increases long-term vulnerability
	**Mental Health Burden**	Co-occurring psychiatric symptom severity	Depression, anxiety, post-traumatic stress disorder	Associated with negative affect, dysregulation, and worse clinical outcomes
	**Socioeconomic Status (SES)**	Access to material and social resources	Income, education, occupational stability	Influences stress exposure, opportunity, and recovery trajectories
Affective/Motivational *(Modulatory)*	**Negative Affect**	Experience of aversive emotional states	Anxiety, depression, and stress symptoms	Increases vulnerability to substance use and relapse
	**Emotional Dysregulation**	Difficulty modulating emotional responses	Reappraisal deficits, heightened reactivity	Promotes maladaptive coping, including substance use
	**Well-Being**	Overall subjective psychological functioning	Positive affect, life satisfaction	Reduced well-being is associated with worse outcomes and recovery difficulty
	**Cue Reactivity**	Heightened response to substance-related cues	Salience attribution, conditioned learning	Triggers craving and increases relapse risk

Different vulnerabilities may shape pathways in distinct ways. For example, impulsivity represents a trait-like vulnerability associated with heightened reward sensitivity and reduced regulatory control, increasing risk for early experimentation and escalation of use [12, 88]. In this case, inherent predispositions and environmental factors interact to influence behavior, suggesting that environmental modification alone may be insufficient to reduce risk. In contrast, trauma-related pathways may be more strongly driven by environmental exposures and learned associations, with downstream effects on stress and reward circuitry [29].In this example, interventions targeting environment, stress regulation, and cognitive-emotional processing may be particularly effective.

Multilevel, circuit-based models further clarify how specific neural systems may integrate these processes within the broader framework. For example, the insula has been proposed as a key region for integrating interoceptive signals into decision-making processes [89]. In this view, internal subjective states are represented in the insula and dynamically influence both motivational and regulatory circuits. These transient subjective states can directly impact cognitive performance and decision-making, as illustrated in the pathway example (Fig. 1), and highlight the importance of incorporating interoceptive and affective processes into comprehensive models of addiction. Such mechanisms also suggest potential intervention targets at the circuit level. In addition to specifying potential pathways, the framework also supports approaches for quantifying heterogeneity and identifying clinically meaningful subgroups (Table 1).

A recent heuristic framework of addiction can serve as a conceptual bridge between circuit-based models and biopsychosocial approaches. The Addictions Neuroclinical Assessment was proposed to capture the addiction cycle domains of incentive salience, executive function, and negative emotionality across substances to better understand why individuals have different pathways and time courses for specific addictions, and why individuals with the same drug addiction respond differently to the same intervention [90]. The authors also provided a framework to understand how multidimensional data could be analyzed in a way that improved precision medicine by using advanced statistical approaches to improve diagnoses of subgroups and to identify specific treatments. However, this neuroclinical assessment is limited in that it treats psychosocial factors as secondary, rather than as vital contributors to the individual pathways.

The biopsychosocial framework envisioned here is one that takes into account many of these nuances, and it positions historical variables as vital factors that disrupt or change development of underlying neurocircuitry, thereby shaping neurocognitive processes that underlie decision-making. In addition, the ability to plan and take action is moderated by current and ongoing emotional and regulatory states of the individual (Fig. 1). Overall, these processes create a maladaptive decision-making framework characterized by rigid, habit-like patterns of thought and behavior. Within this framework, these specific processes can be traced from most to least problematic, identifying changeable circumstances, brain function, and behavior.

Conceptualizing addiction in this way has implications for how treatment targets are identified and prioritized. Despite there being several evidence-based behavioral interventions for addiction [15, 91, 92], efforts typically center on developing pharmacological agents. These compounds can provide biological support towards long-term recovery; however, enduring change typically requires adjustments to environment and behavior, as well. Early efforts included community reinforcement and contingency management, both successful at addressing addiction with contingency management still used today [93, 94]. The community reinforcement idea was novel and strengthened familial and community bonds; yet, it is rarely explicitly employed today, even though it is crucial to improve living situations to achieve positive long-term outcomes [93, 95].

Recently, psychedelic-assisted therapy has been posited as a promising transdiagnostic intervention that is conceptually consistent with multilevel change [96], though underlying mechanisms likely differ between subgroups. Aligning with the biopsychosocial model, results with psychedelic-assisted treatment suggest brain network-level changes in functional connectivity, positive reappraisal of prior adversity, and improved clinical, cognitive, social, and mood outcomes [97–100]. Rapid increases in neuroplasticity provide opportunities for insights, behavior, and brain changes to last longer and increase sustained recovery [101, 102].

## A Systems-Level Understanding of Addiction

Bench-to-bedside expectations have often underperformed across multiple biomedical domains, raising concerns about reproducibility and translation [103–105]. A multilevel framing may better support mechanistic precision while respecting clinical complexity [85, 90].

Over the past several decades, models of addiction have evolved from relatively circumscribed accounts of maladaptive reward learning to increasingly integrative frameworks that attempt to capture the complexity of substance use disorders. Early dopamine-centered models provided critical insights into reinforcement, motivation, and habit formation, and they continue to inform basic neuroscience research. However, their limited success in translating to effective clinical interventions has highlighted the insufficiency of single-circuit explanations for a disorder characterized by marked heterogeneity in presentation, course, and treatment response.

Dual-process and tripartite models, explaining impaired response inhibition, salience attribution, allostasis, and the addiction cycle, substantially expanded explanatory power by incorporating executive dysfunction, negative affect, and relapse dynamics. These models remain foundational and continue to guide contemporary research. Nonetheless, they primarily emphasize neural mechanisms and often treat psychological and social influences as secondary or downstream contributors, rather than as interacting drivers that shape addiction trajectories across individuals and contexts.

Accumulating evidence increasingly supports addiction as a multilevel disorder in which biological vulnerability, cognitive and affective processes, developmental history, and social environment interact dynamically over time. Converging frameworks outside of substance use research further support this multilevel perspective. For example, the Interaction of Person-Affect-Cognition-Execution (I-PACE) model, developed in the context of behavioral addictions, similarly emphasizes dynamic interactions among individual predispositions, affective and cognitive responses, and executive control processes in shaping maladaptive behaviors [106]. Although originally applied to non-substance-related disorders, the I-PACE model reinforces the broader principle that addiction-related behaviors emerge from interacting neurobiological, psychological, and contextual processes over time, aligning with biopsychosocial approaches that emphasize pathway-specific heterogeneity and multilevel intervention targets.

Similarly, for drug addictions, cognitive impairments, psychiatric comorbidities, trauma exposure, and socioeconomic instability are not peripheral features of addiction but core components that influence initiation, maintenance, and recovery. Large-scale network approaches and human neuroimaging studies further underscore that addiction-related dysfunction is distributed across interacting systems rather than localized to a single pathway or region [107–109].

The biopsychosocial perspective offers a comprehensive framework for integrating these findings, yet its complexity has been historically limited in its application to mechanistic research and intervention development. Recent advances in neuroimaging, computational modeling, and multivariate statistical approaches now provide the tools necessary to empirically interrogate biopsychosocial interactions rather than treating them as conceptual abstractions. Such approaches allow for the identification of subtypes, pathways, and modifiable targets that may explain why individuals with similar substance use histories respond differently to treatment; and emerging psychedelic treatments have shown promise for addressing several of these levels of addiction simultaneously.

Importantly, adopting a biopsychosocial perspective does not require abandoning circuit-level or preclinical research; rather, it situates these approaches within a broader systems context that is more consistent with clinical reality. Interventions that appear promising at the neural level may ultimately exert their effects through psychological flexibility, environmental change, or their interaction, underscoring the need for multilevel measurement and theory-driven integration.

## Conclusions

In moving beyond reductionist models, the field is positioned to shift from a search for singular causal mechanisms toward an emphasis on interacting processes and individualized pathways of risk and recovery. Framing addiction as a multilevel disorder may not only improve mechanistic understanding but also provide a more rational foundation for the development, evaluation, and personalization of future interventions.

## Key References

- Nardou R, Sawyer E, Song YJ, Wilkinson M, Padovan-Hernandez Y, de Deus JL, et al. Psychedelics reopen the social reward learning critical period. Nature. Nature Publishing Group; 2023;618:790–8. 10.1038/s41586-023-06204-3.
  - ○A series of preclinical studies demonstrating the strong potential of psychedelics to rewire entrenched neural networks that govern social and reward related behaviors.
- Rust N. Elusive cures: Why neuroscience hasn’t solved brain disorders—and how we can change that. Princeton University Press; 2025.
  - ○A book providing compelling arguments and evidence for the lack of success in developing bench-to-bedside interventions for brain disorders, suggesting a paradigm shift is needed to improve treatment outcomes.

## Data Availability

No datasets were generated or analysed during the current study.

## References

[1] Degenhardt L, Hall W. Extent of illicit drug use and dependence, and their contribution to the global burden of disease. Lancet. 2012;379:55–70. 22225671 10.1016/S0140-6736(11)61138-0

[2] Volkow ND, Koob GF, McLellan AT. Neurobiologic Advances from the Brain Disease Model of Addiction. New Engl J Med Mass Med Soc. 2016;374:363–71. 10.1056/NEJMra1511480 10.1056/NEJMra1511480 PMC6135257 26816013

[3] Ciccarone D. The Rise of Illicit Fentanyls, Stimulants and the Fourth Wave of the Opioid Overdose Crisis. Curr Opin Psychiatry. 2021;34:344–50. 10.1097/YCO.0000000000000717 33965972 10.1097/YCO.0000000000000717 PMC8154745

[4] Friedman J, Montero F, Bourgois P, Wahbi R, Dye D, Goodman-Meza D, et al. Xylazine spreads across the US: A growing component of the increasingly synthetic and polysubstance overdose crisis. Drug Alcohol Depend. 2022;233:109380. 10.1016/j.drugalcdep.2022.109380 35247724 10.1016/j.drugalcdep.2022.109380 PMC9128597

[5] Cano M, Daniulaityte R, Marsiglia F. Xylazine in Overdose Deaths and Forensic Drug Reports in US States, 2019-2022. JAMA Netw Open. 2024;7:e2350630. 10.1001/jamanetworkopen.2023.50630 38180756 10.1001/jamanetworkopen.2023.50630 PMC10770774

[6] O’Donnell J, Tanz LJ, Gladden RM, Davis NL, Bitting J. Trends in and Characteristics of Drug Overdose Deaths Involving Illicitly Manufactured Fentanyls - United States, 2019-2020. MMWR Morb Mortal Wkly Rep. 2021;70:1740–6. 10.15585/mmwr.mm7050e3 34914673 10.15585/mmwr.mm7050e3 PMC8675656

[7] Spencer MR, Garnett MF, Miniño AM. Drug Overdose Deaths in the United States, 2002–2022 [Internet]. National Center for Health Statistics; 2024. Report No.: 491. https://www.cdc.gov/nchs/data/databriefs/db491.pdf

[8] Kinsky S, Houck PR, Mayes K, Loveland D, Daley D, Schuster JM. A comparison of adherence, outcomes, and costs among opioid use disorder Medicaid patients treated with buprenorphine and methadone: A view from the payer perspective. Journal of Substance Abuse Treatment [Internet]. 2019 [cited 2019 June 5]; 10.1016/j.jsat.2019.05.015 10.1016/j.jsat.2019.05.015 31370980

[9] Ma J, Bao Y-P, Wang R-J, Su M-F, Liu M-X, Li J-Q, et al. Effects of medication-assisted treatment on mortality among opioids users: a systematic review and meta-analysis. Mol Psychiatry. 2019;24:1868–83. 10.1038/s41380-018-0094-5. 29934549 10.1038/s41380-018-0094-5

[10] Sordo L, Barrio G, Bravo MJ, Indave BI, Degenhardt L, Wiessing L, et al. Mortality risk during and after opioid substitution treatment: systematic review and meta-analysis of cohort studies. BMJ. 2017;357:j1550. 10.1136/bmj.j1550. 28446428 10.1136/bmj.j1550 PMC5421454

[11] Ramirez A, Arbuckle MR. Synaptic plasticity: the role of learning and unlearning in addiction and beyond. Biol Psychiatry Elsevier. 2016;80:e73-5. 10.1016/j.biopsych.2016.09.002. 10.1016/j.biopsych.2016.09.002 PMC5347979 27697156

[12] Bechara A. Decision making, impulse control and loss of willpower to resist drugs: a neurocognitive perspective. Nat Neurosci Nat Publishing Group. 2005;8:1458–63. 10.1038/nn1584. 10.1038/nn1584 16251988

[13] Redish AD, Jensen S, Johnson A. A unified framework for addiction: vulnerabilities in the decision process. Behav Brain Sci. 2008. 10.1017/S0140525X0800472X. 18662461 10.1017/S0140525X0800472X PMC3774323

[14] Hursh SR, Silberberg A. Economic demand and essential value. Psychol Rev. 2008;115:186–98. 18211190 10.1037/0033-295X.115.1.186

[15] Bickel WK, Snider SE, Quisenberry AJ, Stein JS, Hanlon CA. Competing neurobehavioral decision systems theory of cocaine addiction: from mechanisms to therapeutic opportunities. Prog Brain Res. 2016;223:269–93. 10.1016/bs.pbr.2015.07.009. 26806781 10.1016/bs.pbr.2015.07.009 PMC5495192

[16] Sutherland MT, McHugh MJ, Pariyadath V, Stein EA. Resting state functional connectivity in addiction: lessons learned and a road ahead. Neuroimage. 2012;62:2281–95. 10.1016/j.neuroimage.2012.01.117. 22326834 10.1016/j.neuroimage.2012.01.117 PMC3401637

[17] Brady KT, Tuerk P, Back SE, Saladin ME, Waldrop AE, Myrick H. Combat Posttraumatic Stress Disorder, Substance Use Disorders, and Traumatic Brain Injury. J Addict Med. 2009;3:179–88. 10.1097/ADM.0b013e3181aa244f. 21769015 10.1097/ADM.0b013e3181aa244f PMC4124907

[18] Ursache A, Noble KG. Neurocognitive development in socioeconomic context: multiple mechanisms and implications for measuring socioeconomic status. Psychophysiology. 2016;53:71–82. 10.1111/psyp.12547. 26681619 10.1111/psyp.12547 PMC4685721

[19] Pettinati HM, O’Brien CP, Dundon WD. Current status of co-occurring Mood and Substance Use Disorders: a new therapeutic target. AJP Am J Physiol. 2013;170:23–30. 10.1176/appi.ajp.2012.12010112. 10.1176/appi.ajp.2012.12010112 PMC3595612 23223834

[20] Flagel SB, Robinson TE, Clark JJ, Clinton SM, Watson SJ, Seeman P, et al. An animal model of genetic vulnerability to behavioral disinhibition and responsiveness to reward-related cues: implications for addiction. Neuropsychopharmacology. 2010;35:388–400. 10.1038/npp.2009.142. 19794408 10.1038/npp.2009.142 PMC2794950

[21] Kalivas PW. Neurobiology of cocaine addiction: implications for new pharmacotherapy. Am J Addict. 2007;16:71–8. 10.1080/10550490601184142. 17453607 10.1080/10550490601184142

[22] Heilig M, Epstein DH, Nader MA, Shaham Y. Time to connect: bringing social context into addiction neuroscience. Nat Rev Neurosci. 2016;17:592–9. 10.1038/nrn.2016.67. 27277868 10.1038/nrn.2016.67 PMC5523661

[23] Nutt DJ, Lingford-Hughes A, Erritzoe D, Stokes PRA. The dopamine theory of addiction: 40 years of highs and lows. Nat Rev Neurosci. 2015;16:305–12. 10.1038/nrn3939. 25873042 10.1038/nrn3939

[24] Salamone JD, Correa M. The mysterious motivational functions of mesolimbic dopamine. Neuron. 2012;76:470–85. 10.1016/j.neuron.2012.10.021. 23141060 10.1016/j.neuron.2012.10.021 PMC4450094

[25] Berridge KC, Robinson TE. What is the role of dopamine in reward: hedonic impact, reward learning, or incentive salience? Brain Res Brain Res Rev. 1998;28:309–69. 9858756 10.1016/s0165-0173(98)00019-8

[26] Galea S, Nandi A, Vlahov D. The social epidemiology of substance use. Epidemiol Rev. 2004;26:36–52. 10.1093/epirev/mxh007 15234946 10.1093/epirev/mxh007

[27] Felitti VJ, Anda RF, Nordenberg D, Williamson DF, Spitz AM, Edwards V, et al. Relationship of childhood abuse and household dysfunction to many of the leading causes of death in adults. Am J Prev Med. 1998;14:245–58.10.1016/S0749-3797(98)00017-8 9635069 10.1016/s0749-3797(98)00017-8

[28] Anda RF, Felitti VJ, Bremner JD, Walker JD, Whitfield C, Perry BD, et al. The enduring effects of abuse and related adverse experiences in childhood. A convergence of evidence from neurobiology and epidemiology. Eur Arch Psychiatry Clin Neurosci. 2006;256:174–86. 10.1007/s00406-005-0624-4 16311898 10.1007/s00406-005-0624-4 PMC3232061

[29] Teicher MH, Samson JA, Anderson CM, Ohashi K. The effects of childhood maltreatment on brain structure, function and connectivity. Nat Rev Neurosci. 2016;17:652–66. 10.1038/nrn.2016.111. 27640984 10.1038/nrn.2016.111

[30] Pampel FC, Krueger PM, Denney JT. Socioeconomic disparities in health behaviors. Annu Rev Sociol. 2010;36:349–70. 10.1146/annurev.soc.012809.102529. 21909182 10.1146/annurev.soc.012809.102529 PMC3169799

[31] Kendler KS, Jacobson KC, Prescott CA, Neale MC. Specificity of genetic and environmental risk factors for use and abuse/dependence of cannabis, cocaine, hallucinogens, sedatives, stimulants, and opiates in male twins. Am J Psychiatry. 2003;160:687–95. 12668357 10.1176/appi.ajp.160.4.687

[32] Karkhanis AN, Rose JH, Weiner JL, Jones SR. Early-life social isolation stress increases kappa opioid receptor responsiveness and downregulates the dopamine system. Neuropsychopharmacology. 2016;41:2263–74. 10.1038/npp.2016.21. 26860203 10.1038/npp.2016.21 PMC4946054

[33] Khantzian EJ. The self-medication hypothesis of substance use disorders: a reconsideration and recent applications. Harv Rev Psychiatry. 1997;4:231–44. 10.3109/10673229709030550. 9385000 10.3109/10673229709030550

[34] Fazel S, Khosla V, Doll H, Geddes J. The prevalence of mental disorders among the homeless in western countries: systematic review and meta-regression analysis. PLoS Med. 2008;5:e225. 19053169 10.1371/journal.pmed.0050225 PMC2592351

[35] Castelpietra G, Knudsen AKS, Agardh EE, Armocida B, Beghi M, Iburg KM, et al. The burden of mental disorders, substance use disorders and self-harm among young people in Europe, 1990–2019: findings from the Global Burden of Disease Study 2019. Lancet Reg Health Eur. 2022. 10.1016/j.lanepe.2022.100341. 35392452 10.1016/j.lanepe.2022.100341 PMC8980870

[36] Galea S, Nandi A, Vlahov D. The social epidemiology of substance use. Epidemiol Rev. 2004;26:36–52. 10.1093/epirev/mxh007 15234946 10.1093/epirev/mxh007

[37] Grant BF, Goldstein RB, Saha TD, Chou SP, Jung J, Zhang H, et al. Epidemiology of DSM-5 Alcohol Use Disorder: Results From the National Epidemiologic Survey on Alcohol and Related Conditions III. JAMA Psychiatry. 2015;72:757–66. 10.1001/jamapsychiatry.2015.0584 26039070 10.1001/jamapsychiatry.2015.0584 PMC5240584

[38] Grant BF, Saha TD, Ruan WJ, Goldstein RB, Chou SP, Jung J, et al. Epidemiology of DSM-5 Drug Use Disorder: Results From the National Epidemiologic Survey on Alcohol and Related Conditions–III. JAMA Psychiatry. 2016;73:39–47. 10.1001/jamapsychiatry.2015.2132 26580136 10.1001/jamapsychiatry.2015.2132 PMC5062605

[39] Jacobsen LK, Southwick SM, Kosten TR. Substance use disorders in patients with posttraumatic stress disorder: a review of the literature. J Clin Psychiatry. 2001;62:373–81. 10.1176/appi.ajp.158.8.1184 11481147

[40] Brady KT, Killeen TK, Brewerton T, Lucerini S. Comorbidity of psychiatric disorders and posttraumatic stress disorder. J Clin Psychiatry. 2000;61(Suppl 7):22–32. 10795606

[41] Conway KP, Swendsen J, Husky MM, He J-P, Merikangas KR. Association of Lifetime Mental Disorders and Subsequent Alcohol and Illicit Drug Use: Results From the National Comorbidity Survey-Adolescent Supplement. J Am Acad Child Adolesc Psychiatry. 2016;55:280–8. 10.1016/j.jaac.2016.01.006 27015718 10.1016/j.jaac.2016.01.006

[42] Substance Abuse and Mental Health Services Administration. Key Substance Use and Mental Health Indicators in the United States: Results from the 2022 National Survey on Drug Use and Health [Internet]. Center for Behavioral Health Statistics and Quality, Substance Abuse and Mental Health Services Administration.; 2023. Report No.: HHS Publication No. PEP22-07-01-005, NSDUH Series H-57. https://www.samhsa.gov/data/report/2022-nsduh-annual-national-report

[43] Bourion-Bédès S, Simirea A, Di Patrizio P, Müller O, Clerc-Urmès I, Sy A, et al. Is early outpatient satisfaction with substance use disorder care a predictor of early dropout? Results of the SUBUSQOL cohort. J Subst Abuse Treat. 2020;119:108151. 10.1016/j.jsat.2020.108151 33032861 10.1016/j.jsat.2020.108151

[44] Simpson TL, Goldberg SB, Louden DKN, Blakey SM, Hawn SE, Lott A, et al. Efficacy and acceptability of interventions for co-occurring PTSD and SUD: A meta-analysis. J Anxiety Disord. 2021;84:102490.10.1016/j.janxdis.2021.102490 34763220 10.1016/j.janxdis.2021.102490 PMC8819868

[45] Warden D, Rush AJ, Trivedi MH, Fava M, Wisniewski SR. The STAR*D Project results: a comprehensive review of findings. Curr Psychiatry Rep. 2007;9:449–59. 10.1007/s11920-007-0061-3 18221624 10.1007/s11920-007-0061-3

[46] Mental Illness - National Institute of Mental Health (NIMH.) [Internet]. [cited 2026 Jan 4]. Accessed 4 Jan 2026. https://www.nimh.nih.gov/health/statistics/mental-illness

[47] Verdejo-Garcia A. Chapter 16 - Executive Dysfunction in Addiction. In: Goldberg E, editor. Executive Functions in Health and Disease [Internet]. San Diego: Academic; 2017. pp. 395–403. [cited 2024 Jan 15]. 10.1016/B978-0-12-803676-1.00016-7

[48] Ceceli AO, Huang Y, Kronberg G, McClain N, King SG, Butelman ER, et al. The Impaired Response Inhibition and Salience Attribution Model of Drug Addiction: Recent Neuroimaging Evidence and Future Directions. Annu Rev Psychol. 2025. 10.1146/annurev-psych-040725-025923 41032574 10.1146/annurev-psych-040725-025923 PMC13099233

[49] Verdejo-Garcia A. New insights on neurocognition in cocaine use disorder. Curr Behav Neurosci Rep. 2018;5:232–7. 10.1007/s40473-018-0163-8.

[50] Hou X, Liu R, Zhou Y, Guan L, Zhou J, Liu J, et al. Shared and unique alterations of large-scale network connectivity in drug-free adolescent-onset and adult-onset major depressive disorder. Transl Psychiatry. 2024;14:255. 10.1038/s41398-024-02974-0. 38866779 10.1038/s41398-024-02974-0 PMC11169372

[51] Koob GF. The role of CRF and CRF-related peptides in the dark side of addiction. Brain Res. 2010;1314:3–14. 10.1016/j.brainres.2009.11.008 19912996 10.1016/j.brainres.2009.11.008 PMC2819562

[52] Domínguez-Salas S, Díaz-Batanero C, Lozano-Rojas OM, Verdejo-García A. Impact of general cognition and executive function deficits on addiction treatment outcomes: systematic review and discussion of neurocognitive pathways. Neurosci Biobehav Rev. 2016;71:772–801. 10.1016/j.neubiorev.2016.09.030. 27793597 10.1016/j.neubiorev.2016.09.030

[53] Carli M, Evenden JL, Robbins TW. Depletion of unilateral striatal dopamine impairs initiation of contralateral actions and not sensory attention. Nature. 1985;313:679–82. 10.1038/313679a0. 3974701 10.1038/313679a0

[54] Olds J, Milner P. Positive reinforcement produced by electrical stimulation of septal area and other regions of rat brain. J Comp Physiol Psychol. 1954;47:419–27. 10.1037/h0058775. 13233369 10.1037/h0058775

[55] Wise RA. Dopamine, learning and motivation. Nat Rev Neurosci. 2004;5:483–94. 10.1038/nrn1406. 15152198 10.1038/nrn1406

[56] Everitt BJ, Robbins TW. Neural systems of reinforcement for drug addiction: from actions to habits to compulsion. Nat Neurosci. 2005;8:1481–9. 10.1038/nn1579. 16251991 10.1038/nn1579

[57] Atkinson RC, Herrnstein RJ, Lindzey G, Luce RD. Stevens’ Handbook of Experimental Psychology, Learning and Cognition. Wiley; 1988.

[58] Nesse RM, Berridge KC. Psychoactive drug use in evolutionary perspective. Science. 1997;278:63–6. 10.1126/science.278.5335.63. 9311928 10.1126/science.278.5335.63

[59] Robinson TE, Berridge KC. The neural basis of drug craving: An incentivesensitization theory of addiction. Brain Research Reviews. 1993;18:247–91.10.1016/0165-0173(93)90013-P. 10.1016/0165-0173(93)90013-p 8401595

[60] Berridge KC, Robinson TE. What is the role of dopamine in reward: hedonicimpact, reward learning, or incentive salience? Brain Research Reviews.1998;28:309–69. https://doi.org/10.1016/S0165-0173(98)00019-8 10.1016/S0165-0173(98)00019-8. 10.1016/s0165-0173(98)00019-8 9858756

[61] Peciña S, Smith KS, Berridge KC. Hedonic hot spots in the brain. Neuroscientist. 2006;12:500–11. 10.1177/1073858406293154. 17079516 10.1177/1073858406293154

[62] Schultz W. A neural substrate of prediction and reward. Science. 1997;275:1593–9. 10.1126/science.275.5306.1593. 9054347 10.1126/science.275.5306.1593

[63] Schultz W. Dopamine reward prediction-error signalling: a two-component response. Nat Rev Neurosci. 2016;17:183–95. 10.1038/nrn.2015.26. 26865020 10.1038/nrn.2015.26 PMC5549862

[64] Solinas M, Belujon P, Fernagut PO, Jaber M, Thiriet N. Dopamine and addiction: what have we learned from 40 years of research. J Neural Transm (Vienna). 2019;126:481–516. 10.1007/s00702-018-1957-2 30569209 10.1007/s00702-018-1957-2

[65] Rust N. Elusive cures: why neuroscience hasn’t solved brain disorders—and how we can change that. Princeton University Press; 2025.

[66] Lenoir M, Serre F, Cantin L, Ahmed SH. Intense sweetness surpasses cocaine reward. PLOS ONE Public Libr Sci. 2007;2:e698. 10.1371/journal.pone.0000698. 10.1371/journal.pone.0000698 PMC1931610 17668074

[67] Ahmed SH. Validation crisis in animal models of drug addiction: beyond non-disordered drug use toward drug addiction. Neurosci Biobehav Rev. 2010;35:172–84. 10.1016/j.neubiorev.2010.04.005. 20417231 10.1016/j.neubiorev.2010.04.005

[68] Minozzi S, Amato L, Pani PP, Solimini R, Vecchi S, De Crescenzo F, et al. Dopamine agonists for the treatment of cocaine dependence. Cochrane Database Syst Rev. 2015;2015:CD003352. 10.1002/14651858.CD003352.pub4. 26014366 10.1002/14651858.CD003352.pub4 PMC6999795

[69] Kosten TR, George TP, Kosten TA. The potential of dopamine agonists in drug addiction. Expert Opin Investig Drugs. 2002;11:491–9. 10.1517/13543784.11.4.491. 11922858 10.1517/13543784.11.4.491

[70] Samaha A-N, Seeman P, Stewart J, Rajabi H, Kapur S. Breakthrough dopamine supersensitivity during ongoing antipsychotic treatment leads to treatment failure over time. J Neurosci Soc Neurosci. 2007;27:2979–86. 10.1523/JNEUROSCI.5416-06.2007. 10.1523/JNEUROSCI.5416-06.2007 PMC6672560 17360921

[71] Berridge KC, Robinson TE. Liking. wanting and the Incentive-Sensitization Theory of addiction. Am Psychol. 2016;71:670–9. 10.1037/amp0000059. 27977239 10.1037/amp0000059 PMC5171207

[72] Stewart J, de Wit H, Eikelboom R. The role of unconditioned and conditioned drug effects in the self-administration of opiates and stimulants. Psychol Rev. 1984;91:251–68. 6571424

[73] Menon V. Large-scale brain networks and psychopathology: a unifying triple network model. Trends Cogn Sci. 2011;15:483–506. 10.1016/j.tics.2011.08.003. 21908230 10.1016/j.tics.2011.08.003

[74] Zilverstand A, Huang AS, Alia-Klein N, Goldstein RZ. Neuroimaging impaired response inhibition and salience attribution in human drug addiction: a systematic review. Neuron. 2018;98:886–903. 10.1016/j.neuron.2018.03.048. 29879391 10.1016/j.neuron.2018.03.048 PMC5995133

[75] Goldstein RZ, Volkow ND. Dysfunction of the prefrontal cortex in addiction: neuroimaging findings and clinical implications. Nat Rev Neurosci. 2011;12:652–69. 10.1038/nrn3119. 22011681 10.1038/nrn3119 PMC3462342

[76] Solomon RL, Corbit JD. An opponent-process theory of motivation: I. temporal dynamics of affect. Psychol Rev. 1974;81:119–45. 10.1037/h0036128. 4817611 10.1037/h0036128

[77] Koob GF, Le Moal M. Drug addiction, dysregulation of reward, and allostasis. Neuropsychopharmacology. 2001;24:97–129. 10.1016/S0893-133X(00)00195-0 11120394 10.1016/S0893-133X(00)00195-0

[78] Koob GF. Negative reinforcement in drug addiction: the darkness within. Curr Opin Neurobiol. 2013;23:559–63. 10.1016/j.conb.2013.03.011. 23628232 10.1016/j.conb.2013.03.011

[79] Hyman SE, Malenka RC, Nestler EJ. Neural mechanisms of addiction: the role of reward-related learning and memory. Annu Rev Neurosci. 2006;29:565–98. 10.1146/annurev.neuro.29.051605.113009. 16776597 10.1146/annurev.neuro.29.051605.113009

[80] Goldstein RZ, Volkow ND. Drug addiction and its underlying neurobiological basis: neuroimaging evidence for the involvement of the frontal cortex. Am J Psychiatry. 2002;159:1642–52. 12359667 10.1176/appi.ajp.159.10.1642 PMC1201373

[81] Koob GF, Volkow ND. Neurocircuitry of addiction. Neuropsychopharmacology. 2010;35:217–38. 10.1038/npp.2009.110. 19710631 10.1038/npp.2009.110 PMC2805560

[82] Lüscher C, Malenka RC. Drug-evoked synaptic plasticity in addiction: from molecular changes to circuit remodeling. Neuron. 2011;69:650–63. 10.1016/j.neuron.2011.01.017. 21338877 10.1016/j.neuron.2011.01.017 PMC4046255

[83] Kalivas PW, O’Brien C. Drug addiction as a pathology of staged neuroplasticity. Neuropsychopharmacol Nat Publishing Group. 2008;33:166–80. 10.1038/sj.npp.1301564. 10.1038/sj.npp.1301564 17805308

[84] Marlatt GA. Substance abuse: Implications of a biopsychosocial model for prevention, treatment, and relapse prevention. In: Grabowski J, VandenBos GR, editors. Psychopharmacology: Basic mechanisms and applied interventions [Internet]. Washington: American Psychological Association; 1992. pp. 131–62. [cited 2025 Oct 29]. 10.1037/10114-004

[85] Engel GL. The need for a new medical model: a challenge for biomedicine. Science. 1977;196:129–36. 847460 10.1126/science.847460

[86] McLellan A, Lewis DC, O’Brien CP, Kleber HD. Drug dependence, a chronic medical illness: implications for treatment, insurance, and outcomes evaluation. JAMA. 2000;284:1689–95. 10.1001/jama.284.13.1689. 11015800 10.1001/jama.284.13.1689

[87] Teicher MH. The effects of childhood maltreatment on brain structure. function connectivity.:15. 10.1038/nrn.2016.111 27640984

[88] Dalley JW, Everitt BJ, Robbins TW. Impulsivity, compulsivity, and top-down cognitive control. Neuron. 2011;69:680–94. 10.1016/j.neuron.2011.01.020. 21338879 10.1016/j.neuron.2011.01.020

[89] Noël X, Brevers D, Bechara A. A neurocognitive approach to understanding the neurobiology of addiction. Curr Opin Neurobiol. 2013;23:632–8. 10.1016/j.conb.2013.01.018. 23395462 10.1016/j.conb.2013.01.018 PMC3670974

[90] Kwako LE, Momenan R, Litten RZ, Koob GF, Goldman D. Addictions Neuroclinical Assessment: a neuroscience-based framework for addictive disorders. Biol Psychiatry. 2016;80:179–89. 10.1016/j.biopsych.2015.10.024. 26772405 10.1016/j.biopsych.2015.10.024 PMC4870153

[91] Garland EL, Hanley AW, Kline A, Cooperman NA. Mindfulness-Oriented Recovery Enhancement reduces opioid craving among individuals with opioid use disorder and chronic pain in medication assisted treatment: ecological momentary assessments from a stage 1 randomized controlled trial. Drug Alcohol Depend Irel. 2019;203:61–5. 10.1016/j.drugalcdep.2019.07.007. 10.1016/j.drugalcdep.2019.07.007 PMC6939880 31404850

[92] Kober H, Brewer JA, Height KL, Sinha R. Neural stress reactivity relates to smoking outcomes and differentiates between mindfulness and cognitive-behavioral treatments. NeuroImage. 2017;151:4–13. 10.1016/j.neuroimage.2016.09.042 27693614 10.1016/j.neuroimage.2016.09.042 PMC5373945

[93] Hunt GM, Azrin NH. A community-reinforcement approach to alcoholism. Behav Res Ther. 1973;11:91–104. 4781962 10.1016/0005-7967(73)90072-7

[94] Higgins, Budney AJ, Bickel WK, Foerg FE, Donham R, Badger GJ. Incentives improve outcome in outpatient behavioral treatment of cocaine dependence. Arch Gen Psychiatry. 1994;51:568–76. 10.1001/archpsyc.1994.03950070060011 8031230 10.1001/archpsyc.1994.03950070060011

[95] Meyers RJ, Miller WR, Hill DE, Tonigan JS. Community reinforcement and familytraining (CRAFT): engaging unmotivated drug users in treatment. Journal ofSubstance Abuse. 1998;10:291–308. 10.1016/S08993289(99)00003-6. 10.1016/s0899-3289(99)00003-6 10689661

[96] Bogenschutz MP, Johnson MW. Classic hallucinogens in the treatment of addictions. Prog Neuropsychopharmacol Biol Psychiatry. 2016;64:250–8. 10.1016/j.pnpbp.2015.03.002. 25784600 10.1016/j.pnpbp.2015.03.002

[97] Carhart-Harris RL, Friston KJ. REBUS and the anarchic brain: Toward a unified model of the brain action of psychedelics. Pharmacol Rev. 2019;71:316–44. 10.1124/pr.118.017160. 31221820 10.1124/pr.118.017160 PMC6588209

[98] Davis AK, Barrett FS, May DG, Cosimano MP, Sepeda ND, Johnson MW, et al. Effects of psilocybin-assisted therapy on Major Depressive Disorder: A randomized clinical trial. JAMA Psychiatry. 2021;78:481–9. 10.1001/jamapsychiatry.2020.3285. 33146667 10.1001/jamapsychiatry.2020.3285 PMC7643046

[99] Watts R, Day C, Krzanowski J, Nutt D, Carhart-Harris R. Patients’ accounts of increased connectedness and acceptance after psilocybin for treatment-resistant depression. J Hum Psychol. 2017;57:520–64.

[100] Doss MK, Považan M, Rosenberg MD, Sepeda ND, Davis AK, Finan PH, et al. Psilocybin therapy increases cognitive and neural flexibility in patients with Major Depressive Disorder. Transl Psychiatry. 2021;11:1–10. 10.1038/s41398-021-01706-y. 34750350 10.1038/s41398-021-01706-y PMC8575795

[101] Calder AE, Hasler G. Towards an understanding of psychedelic-induced neuroplasticity. Neuropsychopharmacology. 2023;48:104–12. 10.1038/s41386-022-01389-z. 36123427 10.1038/s41386-022-01389-z PMC9700802

[102] Nardou R, Sawyer E, Song YJ, Wilkinson M, Padovan-Hernandez Y, de Deus JL, et al. Psychedelics reopen the social reward learning critical period. Nature. 2023;618:790–8. 10.1038/s41586-023-06204-3. 37316665 10.1038/s41586-023-06204-3 PMC10284704

[103] Begley CG, Ellis LM. Drug development: raise standards for preclinical cancer research. Nature. 2012;483:531–3. 22460880 10.1038/483531a

[104] Ioannidis JPA. Why most published research findings are false. PLoS Med. 2005;2:e124. 16060722 10.1371/journal.pmed.0020124 PMC1182327

[105] Hyman SE. Revolution stalled. Sci Transl Med. 2012;4:155cm11. 23052291 10.1126/scitranslmed.3003142

[106] Brand M, Wegmann E, Stark R, Müller A, Wölfling K, Robbins TW, et al. The interaction of person-affect-cognition-execution (I-PACE) model for addictive behaviors: update, generalization to addictive behaviors beyond internet-use disorders, and specification of the process character of addictive behaviors. Neurosci Biobehav Rev. 2019;104:1–10. 10.1016/j.neubiorev.2019.06.032. 31247240 10.1016/j.neubiorev.2019.06.032

[107] Lerman C, Gu H, Loughead J, Ruparel K, Yang Y, Stein EA. Large-scale brain network coupling predicts acute nicotine abstinence effects on craving and cognitive function. JAMA Psychiatry. 2014;71:523–30. 10.1001/jamapsychiatry.2013.4091. 24622915 10.1001/jamapsychiatry.2013.4091 PMC4097018

[108] Regier PS, Hager NM, Gawrysiak M, Ehmann S, Ayaz H, Childress AR, et al. Differential large-scale network functional connectivity in cocaine-use disorder associates with drug-use outcomes. Sci Rep. 2025;15:1–11. 10.1038/s41598-025-91465-3. 40113802 10.1038/s41598-025-91465-3 PMC11926260

[109] Ma L, Steinberg JL, Bjork JM, Wang Q, Hettema JM, Abbate A, et al. Altered effective connectivity of central autonomic network in response to negative facial expression in adults with cannabis use disorder. Biol Psychiatry Cogn Neurosci Neuroimaging. 2020;5:84–96. 10.1016/j.bpsc.2019.05.013. 31345781 10.1016/j.bpsc.2019.05.013 PMC8598077

